# Edible and cation-free kiwi fruit derived vesicles mediated EGFR-targeted siRNA delivery to inhibit multidrug resistant lung cancer

**DOI:** 10.1186/s12951-023-01766-w

**Published:** 2023-02-05

**Authors:** Haoying Huang, Xiaohan Yi, Qingyun Wei, Mengyuan Li, Xueting Cai, Yan Lv, Ling Weng, Yujie Mao, Weiwei Fan, Mengmeng Zhao, Zhongpei Weng, Qing Zhao, Kewei Zhao, Meng Cao, Jing Chen, Peng Cao

**Affiliations:** 1 School of Pharmacy, Nanjing University of Chinese Medicine, Nanjing, 210023 China; 2 Affiliated Hospital of Integrated Traditional Chinese and Western Medicine, Nanjing University of Chinese Medicine, Nanjing, 210028 Jiangsu China; 3 Gaoyou Hospital of Traditional Chinese Medicine, Yangzhou, 225600 Jiangsu China; 4 Guangzhou Key Laboratory of Chinese Medicine Research on Prevention and Treatment of Osteoporosis, The Third Affiliated Hospital of Guangzhou University of Chinese Medicine, No.261 and 263, Longxi Avenue, Guangzhou, 510378 China; 5 Zhenjiang Hospital of Chinese Traditional and Western Medicine, Zhenjiang, 212000 China; 6 Haihe Laboratory of Modern Chinese Medicine, Jinghai District, No.10 Poyanghu Road, 301617 Tianjin, China

**Keywords:** Kiwi, Extracellular vesicles, RNA aptamers, Targeted siRNA delivery, Non-small cell lung cancer, Drug resistance

## Abstract

Clinically, activated EGFR mutation associated chemo-drugs resistance has severely threaten NSCLC patients. Nanoparticle based small interfering RNA (siRNA) therapy representing another promising alternative by silencing specific gene while still suffered from charge associated toxicity, strong immunogenicity and poor targetability. Herein, we reported a novel EGFR-mutant NSCLC therapy relying on edible and cation-free kiwi-derived extracellular vesicles (KEVs), which showed sevenfold enhancement of safe dosage compared with widely used cationic liposomes and could be further loaded with Signal Transducer and Activator of Transcription 3 interfering RNA (siSTAT3). siSTAT3 loaded KEVs (STAT3/KEVs) could be easily endowed with EGFR targeting ability (STAT3/EKEVs) and fluorescence by surface modification with tailor-making aptamer through hydrophobic interaction. STAT3/EKEVs with a controlled size of 186 nm displayed excellent stability, high specificity and good cytotoxicity towards EGFR over-expressing and mutant PC9-GR4-AZD1 cells. Intriguingly, the systemic administration of STAT3/EKEVs significantly suppressed subcutaneous PC9-GR4-AZD1 tumor xenografts in nude mice by STAT3 mediated apoptosis. This safe and robust KEVs has emerged as the next generation of gene delivery platform for NSCLC therapy after multiple drug-resistance.

**Graphical Abstract:**

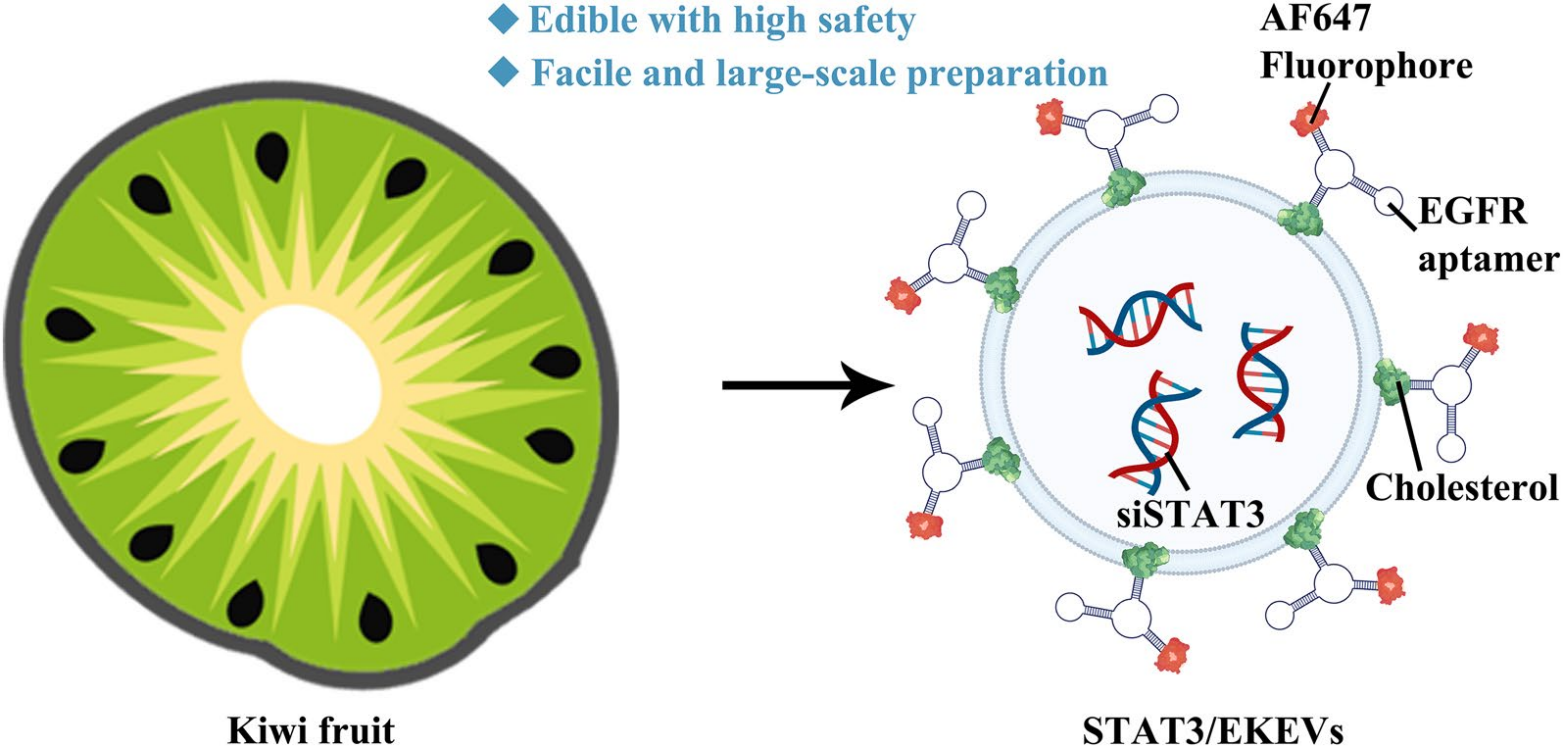

**Supplementary Information:**

The online version contains supplementary material available at 10.1186/s12951-023-01766-w.

## Introduction

Lung cancer constantly took over the throne of global cancer deaths with over 85% of cases currently classified as non-small cell lung cancer (NSCLC) [1–3]. Drug resistance has been widely known as the leading cause to making contribution to the failure of various chemotherapies against NSCLC [4–6]. For instance, Epidermal Growth Factor Receptor (EGFR) tyrosine kinase inhibitors (TKIs) have developed the third generation but will finally make patients progress due to activated mutations in EGFR [7]. Further, such small-molecule inhibitors are notoriously prone to rapid clearance and off-targeting side effects [8], which making it even more challenging to treat EGFR-mutant NSCLC.

Signal Transducer and Activator of Transcription 3 (STAT3) is one of the key downstream signaling mediators of activated EGFR, which was proved to be associated with tumor angiogenesis, cell proliferation, and chemo-resistance [9–11]. Targeting STAT3 could thus provide a novel and promising approach for NSCLC therapy, especially after EGFR mutations [12]. Small interfering ribonucleic acid (siRNA) could selectively inhibit targeted gene expression in the cytoplasm, thus making it promising for various disease treatment, such as cancers, hypercholesterolemia, hepatitis B, acute kidney injury etc [13–16]. While naked siRNA is easily degraded by RNase enzymes in the bloodstream, further making it lose the function [17, 18]. More to the point, siRNA is usually immunogenic and lack of the ability of penetrating through the membrane to enter the targeting cancer cells [18].

Owing to the improved pharmacokinetics and permeability as well as targetability, nanotechnology-based siRNA delivery has shed light on the various cancer therapies [19–22]. Up to now, the most commonly used siRNA delivery systems are still cationic, the majority of which are cationic lipid nanoparticles or liposomes featured with not only efficient loading of siRNA through electrostatic interaction but also promoting cellular internalization and endosomal escape [23–25]. Onpattro was the only one approved siRNA drug delivered by the cationic amino MC3 lipid nanoparticle [26, 27]. However, it was always accompanied with controversial safety issue due to the potential normal cell cytotoxicity and immunogenicity [28, 29]. To solve the problem, its surface charge, size and lipid structure needed to be delicately controlled, which remain complicated [30–33].

**Fig. 1:**
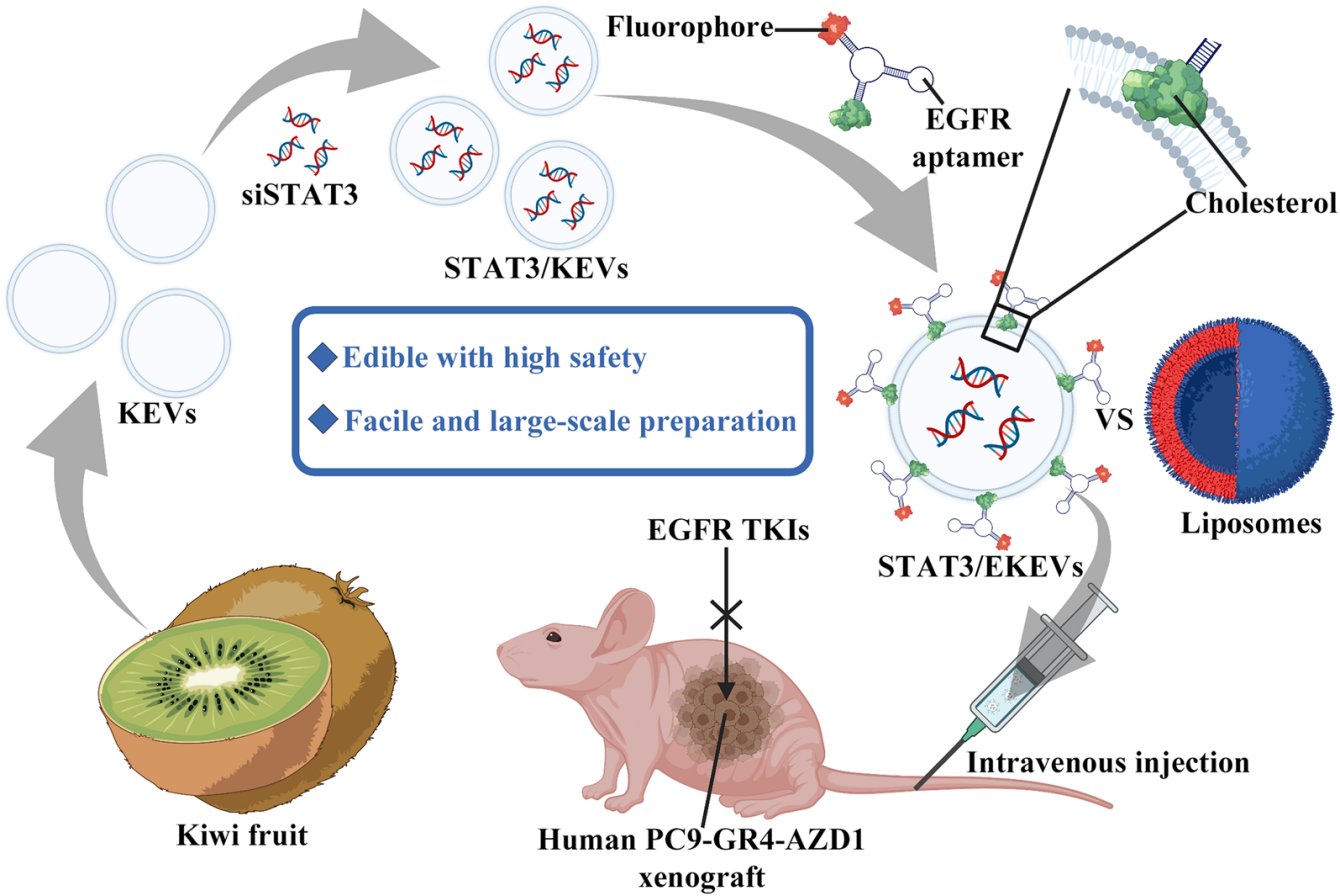
Schematic illustration of siSTAT3 loaded EKEVs (STAT3/EKEVs) for efficient treatment of PC9-GR4-AZD1 NSCLC tumor xenograft

At the same time, with the hollow structure similar to above-mentioned cationic liposomes, edible plants-derived extracellular vesicles (EPEVs) have emerged as new alternative for siRNA delivery [34–36]. For example, grapefruit, ginger and lemon has been reported for making such EPEVs featured with high safety and enviable yields for colon cancer and myeloid leukemia therapy, respectively [37–40]. Here, kiwi-derived extracellular vesicles (KEVs) were designed as siRNA carriers (Fig. 1). To our knowledge, KEVs have not been studied before to treat multidrug resistant lung cancer. Meanwhile, kiwifruit extracts were reported to have broad spectrum anti-tumor effects including lung cancer [41–43]. Here, we reported an innovative and simple approach of the surface modification of KEVs with EGFR aptamer to target EGFR-mutant NSCLC. It should be noted that fluorescent probe could also be tailor-made and integrated into the aptamer, which would allow facile tracking the fate of KEVs in vitro and in vivo.

## Results and discussion

### Characterization of KEVs

**Fig. 2:**
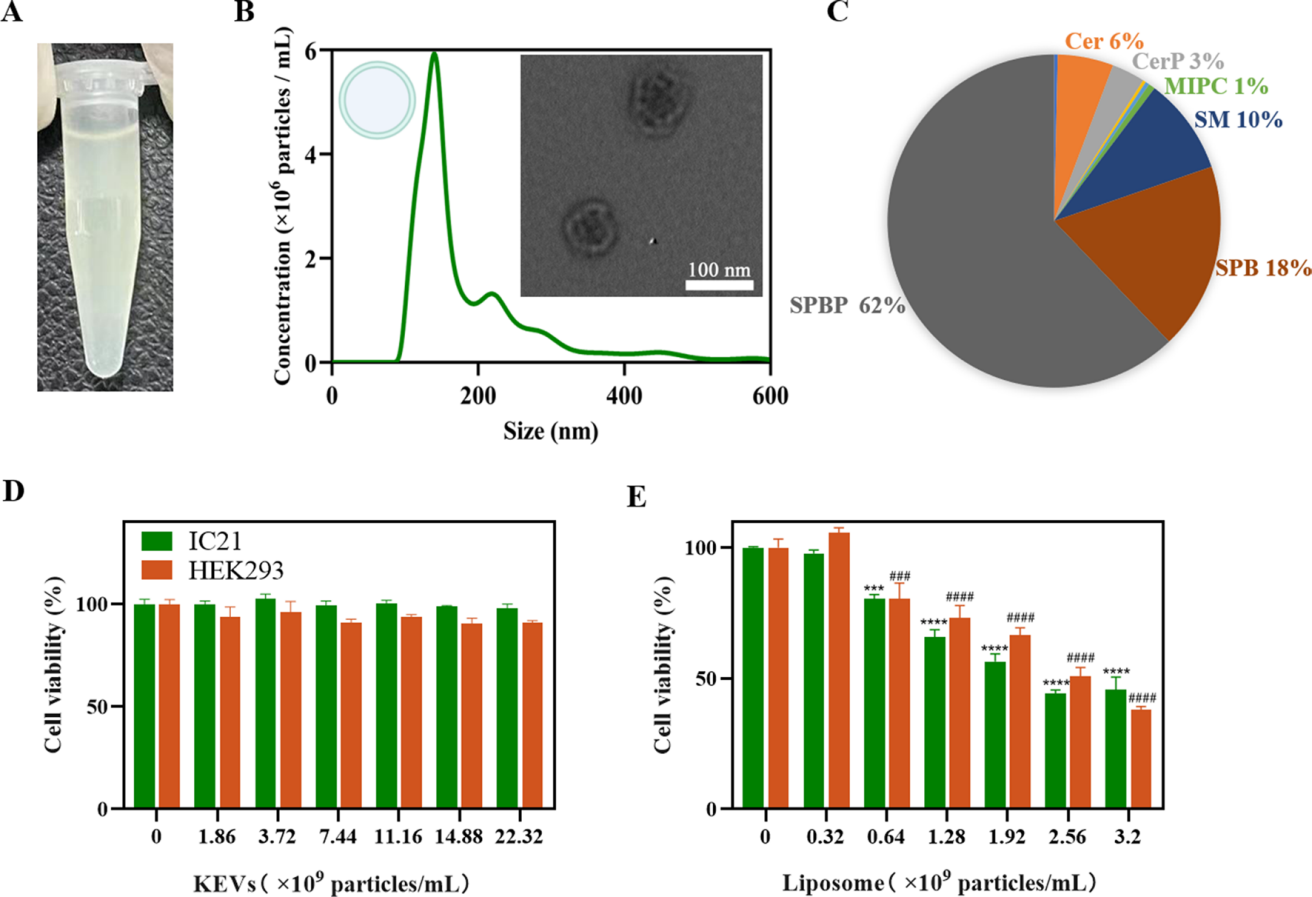
Characterization of KEVs. **A** Picture of KEVs dispersed in PBS. **B** The particle size distribution and representative TEM images of KEVs, respectively. **C** Lipid profiles of KEVs (*SPBP* Sphingoid base-phosphates, *SPB* Sphingoid bases, *SM* Sphingomyelins, *Cer* Ceramides, *CerP* Ceramide-phosphates, *MIPC* Mannosyl-inositolphosphoceramides). Viabilities of IC21 and HEK293 cells after treatment with **D** KEVs and **E** cationic liposomes at different concentrations. One-way ANOVA. ***P < 0.001, ****P < 0.0001, versus the IC21 group. ^###^*P* < 0.001, ^####^*P* < 0.0001, versus the HEK293 group. Error bars represent SEM (n = 3)

KEVs were isolated from Kiwi fruit with a combination of extraction, filtration and differential centrifugation followed by sucrose density gradient ultracentrifugation according to previous reports (Additional file 1: Fig. S1) [44, 45]. It showed good colloidal stability in PBS with the concentration of 7.35 mg/mL (Fig. 2A). Meanwhile, KEVs displayed an average particle size of ~ 198 nm with the spherical morphology (Fig. 2B). The surface charge of KEVs was determined to be − 14.6 mV. As expected, the KEVs exhibited good serum stability with little change in size after 24 h incubation in 10% fetal calf serum at 37 ℃ (Additional file 1: Fig. S2). The total lipid composition of KEVs revealed by LC–MS/MS showed that KEVs mainly contained sphingoid base-phosphates (~ 62%), sphingoid base (~ 18%) and sphingomyelins (~ 10%) (Fig. 2C). Notably, compared with mammalian-derived EVs, KEVs do not require cumbersome cell culture thus the yield is higher. Although remaining concerns, various liposomal therapeutics have been successfully commercialized for cancer and virus treatment till now [46–49]. Thus, cationic liposomes were selected here as a control for in vitro biocompatibility test against mouse peritoneal macrophages cells IC-21 and human embryonic kidney cells HEK293. Interestingly, KEVs showed great biocompatibility (> 90%) even at a high concentration of 22.32 × 10 [9] particles/mL, while cationic liposomes treated cell viability strikingly dropped ~ 50% even at a low concentration of 2.56 × 10^9^ particles/mL (Fig. 2D, E). The safe dose of KEVs was up to sevenfold higher than that of cationic liposomes without showing obvious cytotoxicity, which is consistent with previous reports [50, 51]. In addition, by co-incubating KEVs with PC9-GR4-AZD1, it was found that KEVs can inhibit the cell viability of PC9-GR4-AZD1 (Additional file 1: Fig. S3). When the concentration of KEVs reached to 14.88 × 10^9^ particles/mL, the survival rate of PC9-GR4-AZD1 was 89.6%, which was slightly lower than that incubated in IC21 and HEK293 cells (99.1% and 90.8%) under the same concentration, respectively.

### Characterization of STAT3/EKEVs

**Fig. 3:**
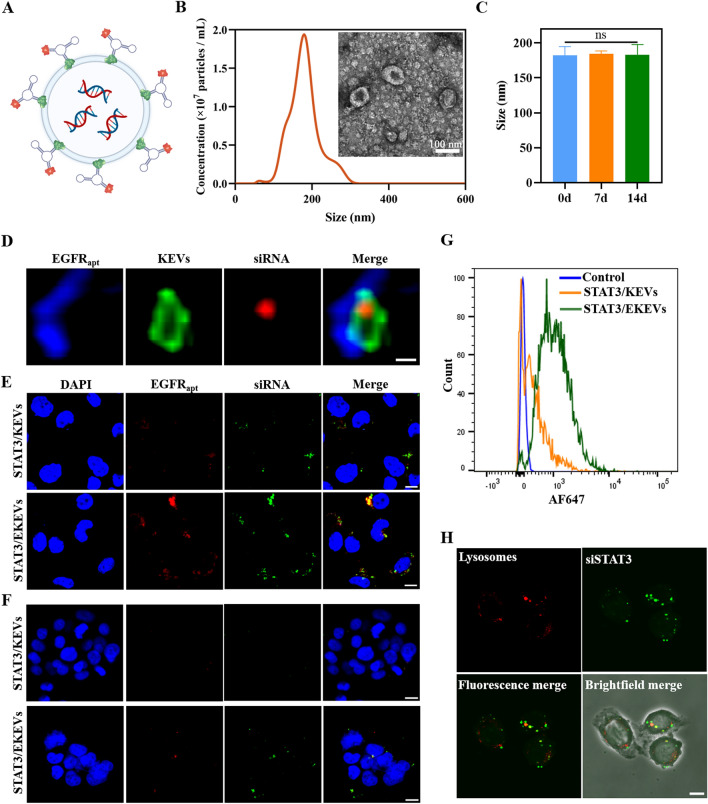
Characterization of STAT3/EKEVs. **A** Structure diagram of STAT3/EKEVs. **B** The particle size distribution and representative TEM images of STAT3/EKEVs, respectively. **C** STAT3/EKEVs size change over 14 days at 4 ℃. One-way ANOVA. *ns* not significant. Error bars represent SEM (n = 3). **D** The detailed structure of STAT3/EKEVs was confirmed using confocal laser scanning microscopy (scale bar: 200 nm). Confocal images of STAT3/EKEVs and STAT3/KEVs in **E** PC9-GR4-AZD1 cells and **F** H520 cells, respectively. (scale bar: 10 μm). **G** Flow cytometry characterization of STAT3/EKEVs and STAT3/KEVs internalization in PC9-GR4-AZD1 cells. **H** Endosome/lysosome escape profiles of STAT3/EKEVs in PC9-GR4-AZD1 cells (scale bar: 10 μm)

EGFR aptamer could be facilely anchored to the surface of KEVs by the hydrophobic interaction of cholesterol and lipids (EGFR aptamer modified KEVs termed as EKEVs) (Fig. 3A). On the other hand, siSTAT3 was loaded into EKEVs by the gentle mix of siRNA and EKEVs (siSTAT3 loaded EKEVs termed as STAT3/EKEVs). It should be emphasized that concentrated STAT3 expression in tumor site of NSCLC patients in early-stage (stage II) was lower than that in late-stage (stage III) featured with a poor 5 year survival time of 15% (Additional file 1: Fig. S4), which was strongly related to the evolving and inevitable drug resistance [52, 53]. Moreover, the easily preformed procedure makes it promising for large scale fabrication, which is essential for prospective clinical application. STAT3/EKEVs had a slightly smaller size of ~ 187 nm (Fig. 3B). The stability of STAT3/EKEVs were evaluated at 4 ℃. As shown in Fig. 3C, the size of STAT3/EKEVs remained similar even after 14 days, indicating its outstanding suspension stability. Meanwhile, the spherical morphology of STAT3/EKEVs maintained similar to that of KEVs. The detailed structure of STAT3/EKEVs was further confirmed using confocal laser scanning microscopy (CLSM) (Fig. 3D). The blue aptamer fluorescence attached to the green fluorescent and ring shaped KEVs which was loaded with Cy3 stained siSTAT3, indicated that aptamer and siRNA was successfully introduced in KEVs with hollow structure. Moreover, approximately 800 pmol siRNA can be loaded per 100 μg KEVs which was confirmed by agarose gel electrophoresis. The loading Efficiency was determined to be about 57.5% and 59.6% for siSTAT3 and siScramble, respectively (Additional file 1: Fig. S5).

The cellular internalization behavior of EKEVs evaluated by CLSM showed strong aptamer fluorescence (AF647) in the cytoplasm of EGFR over-expressing PC9-GR4-AZD1 cells after incubation for 4 h (Fig. 3E), supporting that EKEVs could facilitate targeting to PC9-GR4-AZD1 cells. Expectedly, there was no remarkable difference in EGFR low-expressing H520 cell between EKEVs and 3WJ aptamer modified KEVs (Fig. 3F), further confirmed that EKEVs could actively target to EGFR positive cancer cells. Moreover, the FACS results in consist of CLSM images demonstrated that EKEVs was internalized 2.15 time more than 3WJ aptamer modified KEVs in PC9-GR4-AZD1 cells (Fig. 3G). Endo-lysosomal degradation is an important reason for preventing siRNA from functioning in cells. Live-cell confocal microscopy image (Fig. 3H) showed that STAT3/EKEVs could efficiently release siRNA into cells to avoid phagocytosis and degradation of lysosomes.

### *STAT3/EKEVs inhibition of STAT3 expression *in vitro

**Fig. 4:**
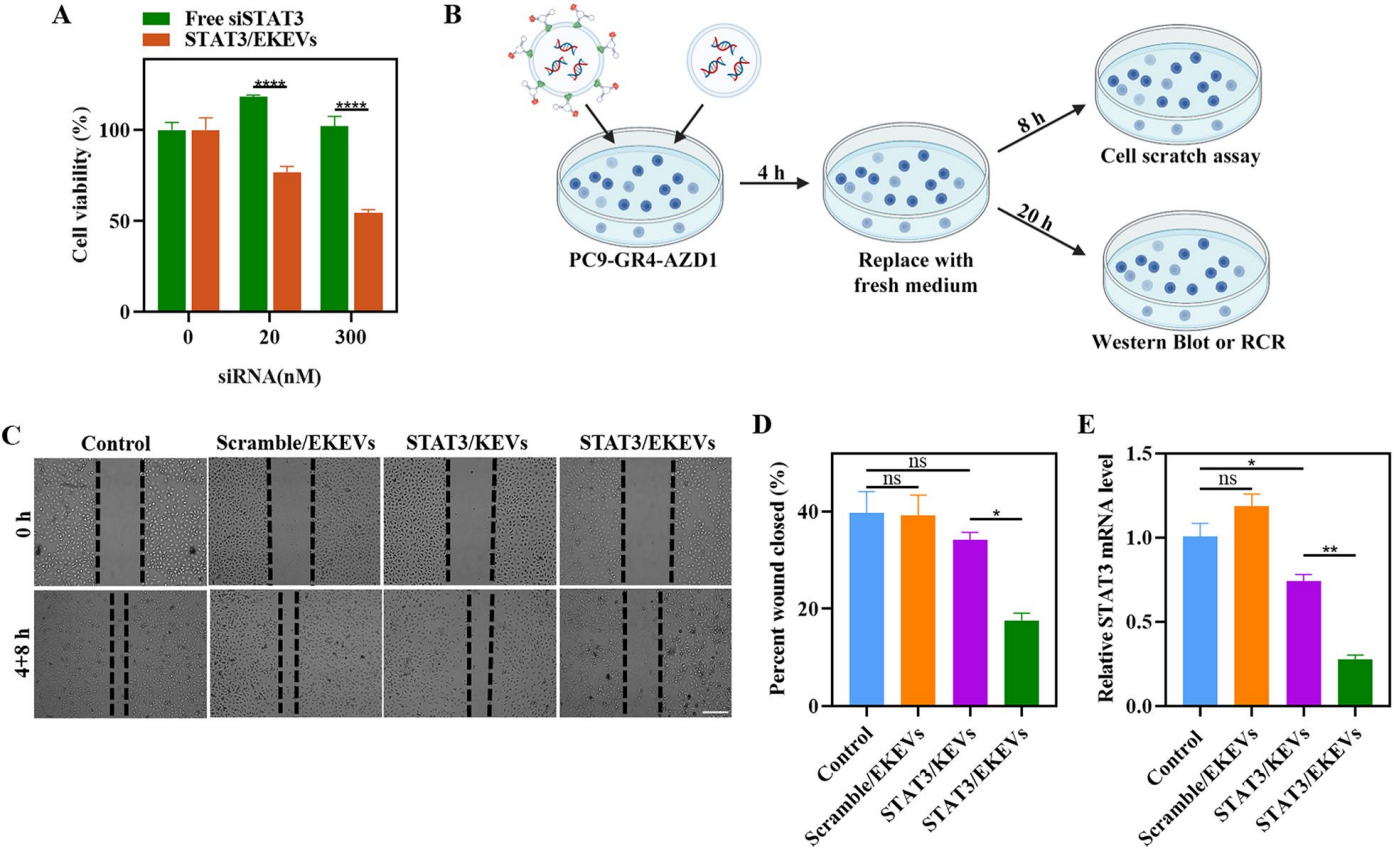
In vitro efficacy. **A** Viabilities of PC9-GR4-AZD1 cells after treatment at different STAT3/EKEVs and free siSTAT3 concentrations. Two-way ANOVA. ****P < 0.0001. Error bars represent SEM (n = 3). **B** Schematic illustration of in vitro experimental procedures. **C** Cell scratch experiments (scale bar: 200 μm) and **D** corresponding quantification. One-way ANOVA. *P < 0.05. *ns* not significant. Error bars represent SEM (n = 3). **E** RT-PCR assays of STAT3 mRNA level in STAT3/EKEVs treated PC9-GR4-AZD1 cells

The inhibition of EGFR was proved to lead to the activation of STAT3 which displayed limited efficacy of EGFR-TKIs against EGFR-mutant lung cancers. Moreover, higher STAT3 mRNA levels in EGFR-TKI-resistant patients were associated with lower survival [54]. Inhibiting STAT3 was known to elicit antitumor activity against NSCLC [55, 56]. The in vitro antitumor activity of STAT3/EKEVs was evaluated by CCK-8 assays in PC9-GR4-AZD1 cells. The results showed that STAT3/EKEVs exhibited a significantly enhanced antitumor activity with increasing dose of STAT3/EKEVs, but free siSTAT3 due to inherent negative charge and large molecular weight did not cause obvious cytotoxicity (Fig. 4A). Cell scratch experiment is widely used method for evaluating cell proliferation and migration ability [57]. Cell scratch as well as corresponding Western Blot and PCR characterization experiments procedures are shown as Fig. 4B. The cell wound was completely healed after treated with PBS or Scramble/EKEVs for 4 + 8 h, supporting that PC9-GR4-AZD1 has great migration ability (Fig. 4C, D). However, cell wound treated with STAT3/EKEVs and STAT3/KEVs exhibited much slower wound recovery, in which STAT3/EKEVs led to minimum wound healing in 4 + 8 h (Fig. 4C, D). STAT3 at mRNA level (Fig. 4E) and protein level (Additional file 1: Fig. S6) in cells was further quantified by qPCR and Western Blot assay, respectively. The data showed the same trends as the cell scratch test.

### In vivo* bio-distribution of STAT3/EKEVs*

**Fig. 5:**
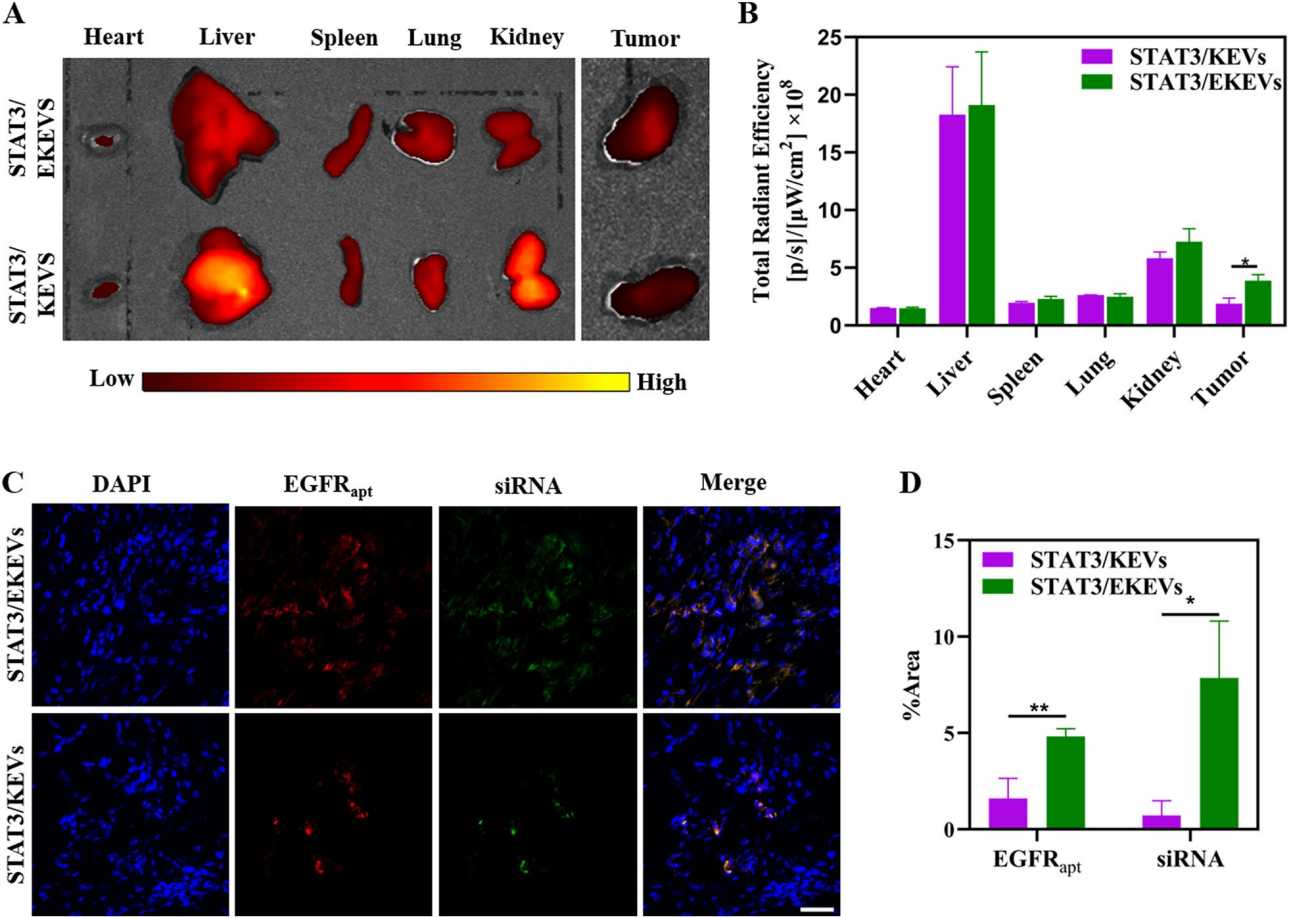
Ex vivo images and bio-distribution of STAT3/EKEVs. **A** Ex vivo fluorescence images and **B** Semi-quantitative analysis of tumors and main organs. Student’s t-test. *P < 0.05. Error bars represent SEM (n = 3). **C** Confocal images of tumors (scale bar: 50 μm) and **D** quantitative analysis of fluorescence distribution in tumors. Student’s t-test. *P < 0.05, **P < 0.01. Error bars represent SEM (n = 3)

In order to further study the targetability of STAT3/EKEVs in vivo, taking nude mice as an example, a PC9-GR4-AZD1 NSCLC subcutaneous xenograft model was established. Further confirmed EGFR expression in tumor site was obviously higher than that in paracancerous tissue (Additional file 1: Fig. S7). Owing to the ligand mediated NSCLC targetability, ex vivo fluorescence images showed that STAT3/EKEVs accumulated to tumor sites more efficiently than STAT3/KEVs, showing a 2.07-fold higher AF647 signal, while without significant enhancement in major organs (Fig. 5A, B). Confocal images of tumor tissues further showed that STAT3/EKEVs could be enriched inside the tumor and could deliver more siRNA into the tumor, with significant statistical differences to STAT3/KEVs (Fig. 5C, D).

### In vivo* antitumor activity of STAT3/EKEVs*

**Fig. 6:**
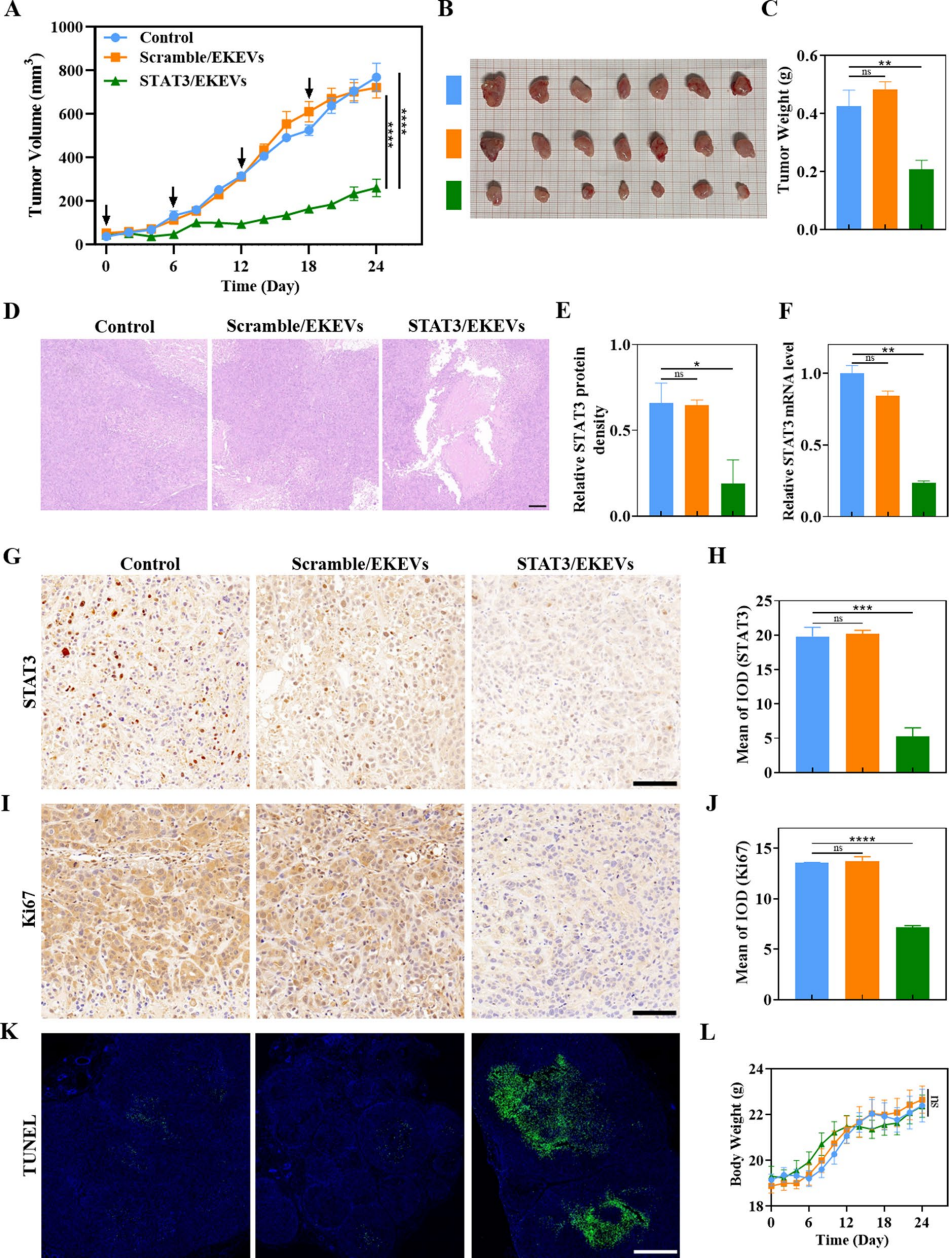
In vivo anti-tumor efficacy of STAT3/EKEVs in PC9-GR4-AZD1 NSCLC subcutaneous xenografts. **A** Tumor growth in different treatment groups. Two-way ANOVA. ****P < 0.0001. Error bars represent SEM (n = 7). **B** Ex vivo photos of tumors. **C** Tumor weight in different treatment groups. One-way ANOVA. **P < 0.01. *ns* not significant. Error bars represent SEM (n = 7). **D** H&E staining images of tumors. **E** STAT3 Western Blot assays of the protein level and **F** RT-PCR assays of gene level after STAT3/EKEVs treatment. One-way ANOVA. *P < 0.05, **P < 0.01. *ns* not significant. Error bars represent SEM (n = 3). **G** STAT3 expression in tumors was determined by IHC. (scale bar: 100 μm). **H** Analysis of STAT3 expression in tumor. One-way ANOVA. ***P < 0.001. *ns* not significant. Error bars represent SEM (n = 3). **I** Ki67 staining images (scale bar: 100 μm) and **J** analysis of Ki67 expression in tumors. One-way ANOVA. ****P < 0.0001. *ns* not significant. Error bars represent SEM (n = 3). **K** TUNEL staining images (scale bar: 1000 μm). **L** Body weight in different treatment groups. Two-way ANOVA. *ns* not significant. Error bars represent SEM (n = 7)

Following encouraging in vitro results, subcutaneous PC9-GR4-AZD1 NSCLC tumor xenografts were used as model to test the in vivo efficacy of STAT3/EKEVs. Mice were intravenously injected with STAT3/EKEVs every 6 days (on day 0, 6, 12, and 18) at a dosage of 240 nmol siSTAT3 equivalents/kg. As expected, STAT3/EKEVs significantly inhibited tumor growth compared with scramble/EKEVs and the PBS control group (Fig. 6A). The photographs of tumors excised on day 24 further corroborated the anti-tumor efficacy of STAT3/EKEVs (Fig. 6B, C). It should be noted that clinically used EGFR tyrosine kinase inhibitors (EGFR TKIs), such as gefitinib, erlotinib, afatinib or osimertinib showed resistance against our tumor model [7]. H&E staining images of tumors showed that STAT3/EKEVs provoked obvious cell necrosis with visible nuclear ablation in contrast to the dense cell arrangement in control groups (Fig. 6D). At the same time, STAT3 was significantly inhibited at the gene level and protein level after treated with STAT3/EKEVs (Fig. 6E, F and Additional file 1: Fig. S8). STAT3 and Ki67 staining displayed less tumor cell proliferation treated with STAT3/EKEVs (Fig. 6G–J). In contrast, TUNEL assays showed that mice treated with STAT3/EKEVs exhibited largest area of tumor cell apoptosis (Fig. 6K). Notably, no significant differences were observed in body weights between the treatment (STAT3/EKEVs) and the negative control (PBS and Scramble/EKEVs) (Fig. 6L), indicating that STAT3/EKEVs have little systemic toxicity.

### Biosafety of STAT3/EKEVs

**Fig. 7:**
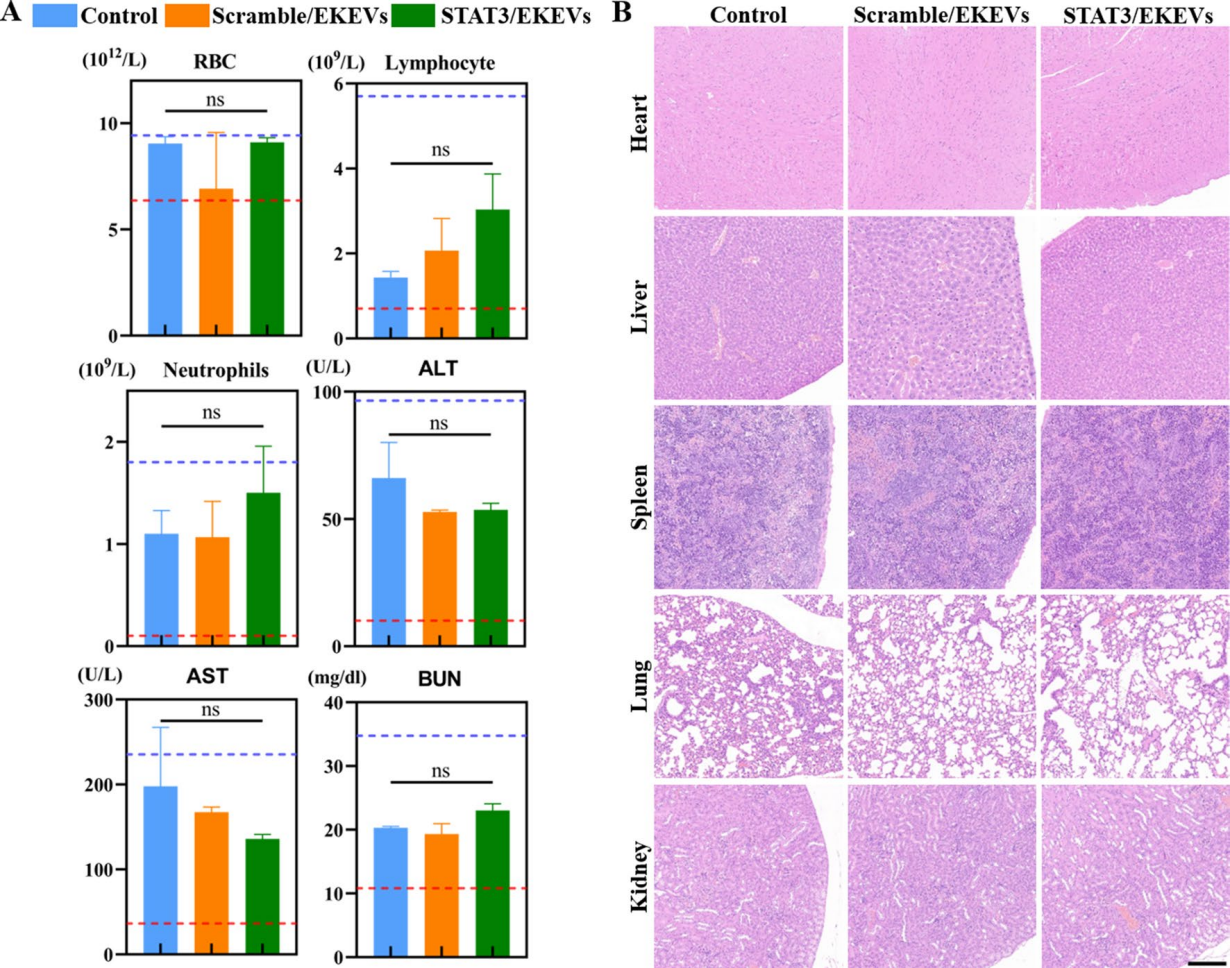
In vivo biosafety evaluation of STAT3/EKEVs. **A** Blood routine, liver function and kidney function. One-way ANOVA. *ns* not significant. Error bars represent SEM (n = 3). **B** H&E staining of different organs. (scale bar: 200 μm)

Inspired by the excellent in vitro biocompatibility data, blood biochemical, hematological analysis and histological examinations were further performed. The results showed that intravenous injection of STAT3/EKEVs did not cause significant pathological changes in blood cells, lymphocytes and neutrophils (Fig. 7A). But the content of lymphocyte and neutrophils still increased a little bit compared with that of the control groups. Thus, the potential anaphylaxis posed by the kiwi fruit should be further tested to confirm the personal safe dosage before application. In contrast, cationic liposome and STAT3 loaded cationic liposome induced obvious tissue toxicity and inflammation (Additional file 1: Fig. S9). Moreover, no organ damage and toxicity were detected in the STAT3/EKEVs group by assessment of liver enzymes, renal function and hematological toxicity (Fig. 7A). In addition, H&E staining showed that STAT3/EKEVs caused no damage to hearts, lungs, livers, spleen and kidneys (Fig. 7B). Collectively, all results indicated that STAT3/EKEVs had no obvious toxic effects both in vitro and in vivo.

## Conclusion

In summary, we have developed edible kiwi-derived extracellular vesicles (KEVs) with much higher safety over traditional used cationic liposomes. More to the point, KEVs with controlled size, excellent stability, surface modification of aptamer and active loading of small interfering RNAs thus could fully release the therapeutic potential of siSTAT3 in PC9-GR4-AZD1 NSCLC subcutaneous xenografts in mice, which could be emerged as a better clinical choice for NSCLC patients after EGFR-TKIs resistance. The work opens up a new avenue of safe and robust RNA delivery system.

## Materials and methods

### Ethics statement, mice and cell lines

All animal experimental protocols were approved by the Institutional Animal Care and Committee of Nanjing University of Chinese Medicine. Female 6-to 8 week-old nude mice were purchased from the Changzhou Cavens Experimental Animal Co., Ltd. (Jiangsu, China). All mice may get free access to food as well as water and were housed in temperature-controlled colony room with 12/12 h dark-light circle. The human non-small cell lung cancer cell line PC9-GR4-AZD1 was generously provided as a gift by Miguel Angel Molina and Rafael Rosell (Pangaea, Barcelona, Spain) [7]. Cells were cultured in RPMI 1640, supplemented with 10% fetal bovine serum, 100 U/mL penicillin, and 100 mg/mL streptomycin (all from Thermo Fisher Scientific, USA). All cells were incubated at 37 °C in a humidified atmosphere with 5% CO_2_.

### RNA sequence

The sequences of all RNA strands (lower-case letters indicate 2′-OME nucleotides) are:

- (1) 3WJ-A-Cholesterol: 5′-uuG ccA uGu GuA uGu GGG /3CholTEG/-3′.
- (2) 3WJ-B: 5′-ccc AcA uAc uuu Guu GAu cc-3′.
- (3) 3WJ-B-EGFR: 5′-ccc AcA uAc uuu Guu GAu ccG ccu uAG uAA cGu Gcu uuG AuG ucG Auu cGA cAG GAG Gc-3′.
- (4) 3WJ-C-AF647: 5′-/5Alex647N/GGA ucA Auc AuG GcA A-3′.

All the RNA oligos were synthesized by Weinabio medicine (Guangdong) Co. LTD.

### Purification and characterization of KEVs

After carefully washing and peeling, edible part of fresh kiwi fruit was put into a juicer to make the juice. Sequentially, differential centrifugation was performed as the following condition: (1) 200 × g, 10 min; (2) 2000 × g, 20 min; (3) 10,000 × g, 30 min to collect the supernatant. The supernatant was further ultracentrifuged at 100,000 × g for 60 min. Bottom sediment was collected and re-suspended in PBS before transferred into sucrose solution (8%, 15%, 30%, 45%, 60%) for density gradient centrifugation (100,000 × g, 60 min). KEVs were finally collected from the band 4 after ultracentrifugation again followed by sterilization with 0.45 μm filter (Additional file 1: Fig.S6) [44]. KEVs were stored at − 80 ℃ for later use. The particle size of KEVs was determined by nanoparticle tracking analysis system (Nanosight3000, Malvern, UK). The morphology of KEVs was characterized by transmission electron microscope (HT-7700, Hitachi, Japan) before stained with 2% uranyl acetate. The concentration of KEVs was quantified by BCA protein assay kit (Beyotime Biotechnology, China) following the manufacturer’s instructions.

### Lipidomic analysis of KEVs

Lipid samples from KEVs were submitted to the Engineering Center of State Ministry of Education for Standardization of Chinese Medicine Processing (Nanjing, China) for lipidomic analysis. In brief, the lipid compositions of KEVs were investigated by using a tof mass spectrometer (AB Sciex TripleTOFTM 5600, AB Sciex, USA). The data were reported as percentages of total signals for the molecular species, which were determined after normalization of the signals to internal standards of the same lipid class.

### RNA-decorated EVs

siRNA was loaded into KEVs (termed as siRNA/KEVs) by mixed with 6.67 μM siRNA and 0.34 μg/μL KEVs according to the method of Exo-Fect^™^ Exosome Transfection Reagent kit. The siRNA/KEVs were removed by centrifugation. Unloaded siRNA in the supernatant was then subjected to agarose electrophoresis and the siRNA loading efficiency was analyzed by Image J. The cholesterol-modified nanoparticles and the KEVs loaded with siRNA were incubated at 37 °C for 45 min, and then incubated on ice for 60 min. The siRNA/EKEVs are purified by ultracentrifugation, and finally re-suspended in 100 μL sterile PBS, and stored at − 80 °C for later use. We defined 3WJ-modified KEVs without EGFR RNA aptamer as 3WJ/KEVs, KEVs with EGFR RNA aptamer modification as STAT3/EKEVs, and encapsulation of siSTAT3 and siScramble as STAT3/EKEVs and Scramble/EKEVs, respectively.

### Characterization of STAT3/EKEVs

The particle size of KEVs was determined by nanoparticle tracking analysis system (Nanosight3000, Malvern, UK). The morphology of KEVs was characterized by transmission electron microscope (HT-7700, Hitachi, Japan) before stained with 2% uranyl acetate. Cy5-labeled siRNA was used to load in DiO-labeled KEVs. AF647-labeled EGFR-apt was used to modify DiO-labeled KEVs. The structure of STAT3/EKEVs imaged with confocal laser scanning microscope (Olympus FV3000, Japan).

### In vitro* targeting of STAT3/EKEVs*

FAM-labeled siRNA was used to load in KEVs. AF647-labeled 3WJ and EGFR-apt were used to modify KEVs according to previous description. For confocal microscopy imaging: PC9-GR4-AZD1 cells (5 × 10^5^/well) were seeded in 24-well plate (Thermo Fisher Scientific, USA) and cultured overnight at 37 ℃. The media was then replaced with fresh culture media containing STAT3/KEVs, STAT3/EKEVs (10 μg/mL). After incubation for 12 h, the cells were fixed with 4% paraformaldehyde for 10 min and dehydrated with acetone at – 20 ℃ for 5 min after rinsed with fresh PBS for three times. 200 μL of 4′,6-Diamidino-2-Phenylindole (DAPI, Beyotime Biotechnology, China) was added and incubated for 30 min to stain the nucleus. Finally, cells were coverslip-mounted with mounting medium and imaged with confocal laser scanning microscope (Olympus FV10i, Japan).

For flow cytometry measurement, PC9-GR4-AZD1 cells (1 × 10^6^/well) were seeded in 6-well plate (Thermo Fisher Scientific, USA). The media was then replaced with fresh culture media containing STAT3/KEVs and STAT3/EKEVs (10 μg/mL). After incubation for 12 h, single-cell suspensions of treated macrophages were prepared in staining buffer. Stained cells were analyzed on a FACS Aria II Flow Cytometer (BD Biosciences, USA). Data analysis was performed using Flow Jo software (BD Biosciences, USA).

### Wound-healing assays

PC9-GR4-AZD1 cells (5 × 10^5^/well) were seeded in 6-well plate and cultured overnight at 37 ℃. Then the 6-well plate was scratched vertically with a 10 μL pipette tip. PBS, STAT3/EKEVs, STAT3/KEVs and Scramble/KEVs (300 nM) were incubated for 4 h. After replaced with fresh medium, the cells were further cultured for 8 h. Images were obtained by MShot Image Analysis System. The average of the healed wound area was measured by comparing 0 h and 12 h using Image J.

### Western blot and antibodies

PC9-GR4-AZD1 cells and tissues were collected and lysed with Radio Immunoprecipitation Assay buffer (Sigma-Aldrich, USA) containing protease inhibitor cocktail (Roche, CH). Primary antibodies used for western blot analysis were STAT3 Mouse mAb (#9139, Cell Signaling Technology, USA) and GAPDH Mouse mAb (#5174, Cell Signaling Technology, USA), respectively.

### Cytotoxicity assay

The cytotoxicity of KEVs, cationic liposomes and STAT3/EKEVs were evaluated with CCK8 assay kit (Med Chem Express, USA). Mouse peritoneal macrophages cells IC-21、human embryonic kidney cells HEK293 and human non-small cell lung cancer cells PC9-GR4-ZD1 cells (5 × 10^3^/well) were seeded in 96-well plates and cultured overnight at 37 ℃. The concentrations of cationic liposomes (Yisheng 40802ES02, China) and KEVs as well as STAT3/EKEVs treated for cells were ranged from 0 to 3.2 × 10^9^ particles/mL, 0 to 22.32 × 10^9^ particles/mL and 0 to 300 nM, respectively. Determination of NP concentration by NTA (Nanosight3000, Malvern, UK). After 48 h, the cell survival rate was analyzed by CCK8 assay on a microplate reader (VARIOSKAN FLASH, Thermo Fisher Scientific, USA).

### Lysosomal colocalization assay

To examine whether STAT3/EKEVs can escape the lysosome-mediated exocytosis pathway, PC9-GR4-AZD1 cells were incubated with 100 nM Lyso-Tracker Red (L8010, Solarbio, China) for 10 min, followed by co-incubation with STAT3/EKEVs (50 nM) for 60 min at 37 ℃. Finally, cells were coverslip-mounted with mounting medium and imaged with confocal laser scanning microscope (Olympus FV10i, Japan).

### Animal experiments

Subcutaneous human non-small cell lung tumor xenografts were established by inoculating PC9-GR4-AZD1 cells (2 × 10^6^ cells/mouse) to the right flanks of female nude mice (6–8 weeks old). The tumor size was measured using digital calipers and tumor volume was calculated using the following equation: V = (length × width^2^)/2. After 24 days, the mice were arbitrarily divided into three groups (n = 7) and treated with intravenous injection of Scramble/EKEVs and STAT3/EKEVs (30 mg KEVs loading 240 nmol siRNA equivalents /kg) via tail vein every 6 days. PBS was used as the negative control. Tumor size and body weight were monitored every 2 days. At the end of the treatment, mice of all groups were sacrificed, and the tumors and main organs were excised for further analysis.

To test the safety properties of conventional cationic liposomes, the blood of the mice were collected 24 h after intravenous injection of cationic liposome and STAT3 loaded cationic liposome, respectively. The blood was then used for blood biochemical detection.

### RNA isolations and real-time PCR

Total RNA was isolated from PC9-GR4-AZD1 cells after treated with TRIzol reagent (Invitrogen, USA) for 5 min. The total RNA was reverse transcribed using HiScript III RT SuperMix for qPCR (+ gDNA wiper) (Vazyme Biotech, China) analysis, ChamQ Universal SYBR qPCR Master Mix (Vazyme Biotech, China). Real-time PCR was performed using 7500 Real-Time PCR System (Applied Biosystems, USA).

### Immunohistochemistry analysis

The tumors and main organs were fixed with 4% paraformaldehyde solution and embedded in paraffin. The sliced organ tissues mounted on the glass slides were stained by hematoxylin and eosin (H&E) and observed by an optical microscope (Olympus, Japan) to analyze microscopic pathological changes. STAT3 Mouse mAb (#9139, Cell Signaling Technology, USA) and Ki67 (GB111499, Service Bio, China) were used for staining in order to evaluate the infiltration of STAT3, Ki67 in tumors. Data analysis was performed using image pro plus6 software (Media Cybernetics, USA).

### TUNEL

Tumor were de-paraffinized, and apoptotic cells were detected by immunofluorescence TUNEL (terminal deoxynucleotidyl transferase-mediated dUTP-biotin nick end labeling) assay using the In Situ Cell Death Detection Kit (GB1501, Service Bio, China). Images were acquired using microscope (NIKON DS-U3, Nikon, Japan).

### Safety evaluation

After the animal experiment, the blood and various tissues of the mice were collected, the blood was used for blood biochemical detection, and the tissues and organs were used for H&E staining detection. The blood biochemical detection and H&E staining were performed by Wuhan servicebio technology Co. LTD.

### Bioinformatics analysis

The data of STAT3 expression level in the NSCLC were obtained from CPTAC_2020 cohort data according to the previous report and analyzed by the Mann–Whitney U test with the one tailed method using the R programming language [52].

### Statistics

Each experiment was repeated at least three times with triplication for each sample tested. The results were presented as mean ± standard deviation, unless otherwise indicated. Statistical mean differences were evaluated using unpaired Student’s t-test, One-way ANOVA or Two-way ANOVA with GraphPad software (P value adjusted for multiple comparisons by Holm’s procedure) with R software, and *P* < 0.05 was considered statistically significant. (*P < 0.05, **P < 0.01, ***P < 0.001, ****P < 0.0001, ^#^*P* < 0.05, ^##^*P* < 0.01 ^###^*P* < 0.001, ^####^*P* < 0.0001).

## Supplementary Information

**Additional file 1: Figure S1.** TEM image of each band after sucrose density gradient centrifugation. Transmission electron microscope observation map of each ban. **Figure S2.** Average size change of the KEVs before and after incubation in 10% fetal calf serum at 37℃ for 24h. **Figure S3.** Viabilities of PC9-GR4-AZD1 cells after treatment with KEVs at different concentrations. **Figure S4.** Early and advanced STAT3 expression of NSCLC. Late-stage (stage III) display higher STAT3 expression level than early-stage (stage II) in the NSCLC. **Figure S5.** Characterization of siRNA loading efficiency. **Figure S6.** STAT3 expression in PC9-GR4-AZD1 cells. (A) The protein level of STAT3 was measured by Western Blot assay. (B) Analysis of the protein expression showed that STAT3/EKNPs treatment significantly reduced the expression of STAT3. **Figure S7.** Expression of EGFR in PC9-GR4-AZD1 NSCLC subcutaneous xenografts and paracancerous tissue. The protein level of EGFR was measured by Western Blot assay. **Figure S8.** STAT3 expression in PC9-GR4-AZD1 NSCLC subcutaneous xenografts. The protein level of STAT3 was measured by Western blot assay. **Figure S9.** Blood routine, liver function and kidney function of cationic liposome and STAT3 loaded cationic liposome treated mice, respectively. One-way ANOVA. ns, not significant. Error bars represent SEM (PBS group, n=3; Liposome group, n = 4; STAT3/Liposome group, n=5).

## Data Availability

The datasets used and/or analyzed during the current study are available from the corresponding author on reasonable request.

## References

[1] Friedlaender A, Addeo A, Russo A, Gregorc V, Cortinovis D, Rolfo CD (2020). Targeted therapies in early stage NSCLC: hype or hope?. Int J Mol Sci.

[2] Sung H, Ferlay J, Siegel RL, Laversanne M, Soerjomataram I, Jemal A, Bray F (2021). Global cancer statistics 2020: GLOBOCAN estimates of incidence and mortality worldwide for 36 cancers in 185 countries. CA Cancer J Clin.

[3] Chen Z, Fillmore CM, Hammerman PS, Kim CF, Wong KK (2014). Non-small-cell lung cancers: a heterogeneous set of diseases. Nat Rev Cancer.

[4] Yang Z, Tam KY (2018). Combination strategies using EGFR-TKi in NSCLC therapy: learning from the gap between pre-clinical results and clinical outcomes. Int J Biol Sci.

[5] Boumahdi S, de Sauvage FJ (2020). The great escape: tumour cell plasticity in resistance to targeted therapy. Nat Rev Drug Discov.

[6] Li H, Wei W, Xu H (2022). Drug discovery is an eternal challenge for the biomedical sciences. Acta Materia Medica.

[7] Bertran-Alamillo J, Cattan V, Schoumacher M, Codony-Servat J, Giménez-Capitán A, Cantero F, Burbridge M, Rodríguez S, Teixidó C, Roman R, Castellví J (2019). AURKB as a target in non-small cell lung cancer with acquired resistance to anti-EGFR therapy. Nat Commun.

[8] Nigro A, Ricciardi L, Salvato I, Sabbatino F, Vitale M, Crescenzi MA, Montico B, Triggiani M, Pepe S, Stellato C, Casolaro V (2019). Enhanced expression of CD47 is associated with off-target resistance to tyrosine kinase inhibitor gefitinib in NSCLC. Front Immunol.

[9] Fan J, Xu G, Chang Z, Zhu L, Yao J (2020). miR-210 transferred by lung cancer cell-derived exosomes may act as proangiogenic factor in cancer-associated fibroblasts by modulating JAK2/STAT3 pathway. Clin Sci.

[10] Zheng Q, Dong H, Mo J, Zhang Y, Huang J, Ouyang S, Shi S, Zhu K, Qu X, Hu W, Liu P (2021). A novel STAT3 inhibitor W2014-S regresses human non-small cell lung cancer xenografts and sensitizes EGFR-TKI acquired resistance. Theranostics.

[11] Lee HJ, Zhuang G, Cao Y, Du P, Kim HJ, Settleman J (2014). Drug resistance via feedback activation of stat3 in oncogene-addicted cancer cells. Cancer Cell.

[12] Mohrherr J, Uras IZ, Moll HP, Casanova E (2020). STAT3: versatile functions in non-small cell lung cancer. Cancers.

[13] Draz MS, Fang BA, Zhang P, Hu Z, Gu S, Weng KC, Gray JW, Chen FF (2014). Nanoparticle-mediated systemic delivery of siRNA for treatment of cancers and viral infections. Theranostics.

[14] Lamb YN (2021). Inclisiran: first approval. Drugs.

[15] Yuen MF, Schiefke I, Yoon JH, Ahn SH, Heo J, Kim JH, Lik Yuen Chan H, Yoon KT, Klinker H, Manns M, Petersen J (2020). RNA interference therapy with ARC-520 results in prolonged hepatitis b surface antigen response in patients with chronic hepatitis B infection. Hepatology.

[16] Alidori S, Akhavein N, Thorek DL, Behling K, Romin Y, Queen D, Beattie BJ, Manova-Todorova K, Bergkvist M, Scheinberg DA, McDevitt MR (2016). Targeted fibrillar nanocarbon RNAi treatment of acute kidney injury. Sci Transl Med.

[17] Lu Y, Li J, Su N, Lu D (2018). The mechanism for siRNA transmembrane assisted by PMAL. Molecules.

[18] Arnold AE, Czupiel P, Shoichet M (2017). Engineered polymeric nanoparticles to guide the cellular internalization and trafficking of small interfering ribonucleic acids. J Control Release.

[19] Subhan MA, Torchilin VP (2019). Efficient nanocarriers of siRNA therapeutics for cancer treatment. Transl Res.

[20] Park J, Park J, Pei Y, Xu J, Yeo Y (2016). Pharmacokinetics and biodistribution of recently-developed siRNA nanomedicines. Adv Drug Deliv Rev.

[21] Barua S, Mitragotri S (2014). Challenges associated with penetration of nanoparticles across cell and tissue barriers: a review of current status and future prospects. Nano Today.

[22] Markman JL, Rekechenetskiy A, Holler E, Ljubimova JY (2013). Nanomedicine therapeutic approaches to overcome cancer drug resistance. Adv Drug Deliv Rev.

[23] Charbe NB, Amnerkar ND, Ramesh B, Tambuwala MM, Bakshi HA, Aljabali AAA, Khadse SC, Satheeshkumar R, Satija S, Metha M, Chellappan DK (2020). Small interfering RNA for cancer treatment: overcoming hurdles in delivery. Acta Pharm Sin B.

[24] Mainini F, Eccles MR (2020). Lipid and polymer-based nanoparticle siRNA delivery systems for cancer therapy. Molecules.

[25] Subhan MA, Torchilin VP (2020). siRNA based drug design, quality, delivery and clinical translation. Nanomedicine.

[26] Akinc A, Maier MA, Manoharan M, Fitzgerald K, Jayaraman M, Barros S, Ansell S, Du X, Hope MJ, Madden TD, Mui BL (2019). The onpattro story and the clinical translation of nanomedicines containing nucleic acid-based drugs. Nat Nanotechnol.

[27] Jiang T, Qiao Y, Ruan W, Zhang D, Yang Q, Wang G, Chen Q, Zhu F, Yin J, Zou Y, Qian R (2021). Cation-free sirna micelles as effective drug delivery platform and potent RNAI nanomedicines for glioblastoma therapy. Adv Mater.

[28] Zuckerman JE, Gritli I, Tolcher A, Heidel JD, Lim D, Morgan R, Chmielowski B, Ribas A, Davis ME, Yen Y (2014). Correlating animal and human phase Ia/Ib clinical data with CALAA-01, a targeted, polymer-based nanoparticle containing siRNA. Proc Natl Acad Sci USA.

[29] Zipkin M (2020). Big pharma buys into exosomes for drug delivery. Nat Biotechnol.

[30] Eygeris Y, Gupta M, Kim J, Sahay G (2022). Chemistry of lipid nanoparticles for RNA delivery. Acc Chem Res.

[31] Merino M, Zalba S, Garrido MJ (2018). Immunoliposomes in clinical oncology: state of the art and future perspectives. J Control Release.

[32] Wang N, Chen M, Wang T (2019). Liposomes used as a vaccine adjuvant-delivery system: from basics to clinical immunization. J Control Release.

[33] Inglut CT, Sorrin AJ, Kuruppu T, Vig S, Cicalo J, Ahmad H, Huang HC (2020). Immunological and toxicological considerations for the design of liposomes. Nanomaterials.

[34] Alfieri M, Leone A, Ambrosone A (2021). Plant-derived nano and microvesicles for human health and therapeutic potential in nanomedicine. Pharmaceutics.

[35] Urzì O, Raimondo S, Alessandro R (2021). Extracellular vesicles from plants: current knowledge and open questions. Int J Mol Sci.

[36] Karamanidou T, Tsouknidas A (2021). Plant-derived extracellular vesicles as therapeutic nanocarriers. Int J Mol Sci.

[37] Wang Q, Zhuang X, Mu J, Deng ZB, Jiang H, Zhang L, Xiang X, Wang B, Yan J, Miller D, Zhang HG (2013). Delivery of therapeutic agents by nanoparticles made of grapefruit-derived lipids. Nat Commun.

[38] Zhang M, Viennois E, Prasad M, Zhang Y, Wang L, Zhang Z, Han MK, Xiao B, Xu C, Srinivasan S, Merlin D (2016). Edible ginger-derived nanoparticles: a novel therapeutic approach for the prevention and treatment of inflammatory bowel disease and colitis-associated cancer. Biomaterials.

[39] Ju S, Mu J, Dokland T, Zhuang X, Wang Q, Jiang H, Xiang X, Deng ZB, Wang B, Zhang L, Roth M (2013). Grape exosome-like nanoparticles induce intestinal stem cells and protect mice from DSS-induced colitis. Mol Ther.

[40] Raimondo S, Saieva L, Cristaldi M, Monteleone F, Fontana S, Alessandro R (2018). Label-free quantitative proteomic profiling of colon cancer cells identifies acetyl-CoA carboxylase alpha as antitumor target of citrus limon-derived nanovesicles. J Proteomics.

[41] Motohashi N, Shirataki Y, Kawase M, Tani S, Sakagami H, Satoh K, Kurihara T, Nakashima H, Mucsi I, Varga A, Molnár J (2002). Cancer prevention and therapy with kiwifruit in Chinese folklore medicine: a study of kiwifruit extracts. J Ethnopharmacol.

[42] Lippi G, Mattiuzzi C (2020). Kiwifruit and cancer: an overview of biological evidence. Nutr Cancer.

[43] Kou L, Zhu Z, Redington C, Bai Q, Wakefield M, Lequio M, Fang Y (2021). Potential use of kiwifruit extract for treatment of melanoma. Med Oncol.

[44] Cao M, Yan H, Han X, Weng L, Wei Q, Sun X, Lu W, Wei Q, Ye J, Cai X, Hu C (2019). Ginseng-derived nanoparticles alter macrophage polarization to inhibit melanoma growth. J Immunother Cancer.

[45] Han X, Wei Q, Lv Y, Weng L, Huang H, Wei Q, Li M, Mao Y, Hua D, Cai X, Cao M (2022). Ginseng-derived nanoparticles potentiate immune checkpoint antibody efficacy by reprogramming the cold tumor microenvironment. Mol Ther.

[46] Hu M, Zhang J, Kong L, Yu Y, Hu Q, Yang T, Wang Y, Tu K, Qiao Q, Qin X, Zhang Z (2021). Immunogenic hybrid nanovesicles of liposomes and tumor-derived nanovesicles for cancer immunochemotherapy. ACS Nano.

[47] Zhang Y, Zhang L, Hu Y, Jiang K, Li Z, Lin YZ, Wei G, Lu W (2018). Cell-permeable NF-κB inhibitor-conjugated liposomes for treatment of glioma. J Control Release.

[48] Huang H, Zhang C, Yang S, Xiao W, Zheng Q, Song X (2021). The investigation of mRNA vaccines formulated in liposomes administrated in multiple routes against SARS-CoV-2. J Control Release.

[49] Persano S, Guevara ML, Li Z, Mai J, Ferrari M, Pompa PP, Shen H (2017). Lipopolyplex potentiates anti-tumor immunity of mRNA-based vaccination. Biomaterials.

[50] Ozpolat B, Sood AK, Lopez-Berestein G (2014). Liposomal siRNA nanocarriers for cancer therapy. Adv Drug Deliv Rev.

[51] He K, Tang M (2018). Safety of novel liposomal drugs for cancer treatment: advances and prospects. Chem Biol Interact.

[52] Gillette MA, Satpathy S, Cao S, Dhanasekaran SM, Vasaikar SV, Krug K, Petralia F, Li Y, Liang WW, Reva B, Krek A (2020). Proteogenomic characterization reveals therapeutic vulnerabilities in lung adenocarcinoma. Cell.

[53] Durm GA, Jabbour SK, Althouse SK, Liu Z, Sadiq AA, Zon RT, Jalal SI, Kloecker GH, Williamson MJ, Reckamp KL, Langdon RM (2020). A phase 2 trial of consolidation pembrolizumab following concurrent chemoradiation for patients with unresectable stage III non-small cell lung cancer: hoosier cancer research network LUN 14–179. Cancer.

[54] Chaib I, Karachaliou N, Pilotto S, Codony Servat J, Cai X, Li X, Drozdowskyj A, Servat CC, Yang J, Hu C, Cardona AF (2017). Co-activation of STAT3 and YES-associated protein 1 (YAP1) pathway in EGFR-mutant NSCLC. J Natl Cancer Inst.

[55] Pore N, Wu S, Standifer N, Jure-Kunkel M, de Los Reyes M, Shrestha Y, Halpin R, Rothstein R, Mulgrew K, Blackmore S, Martin P (2021). Resistance to durvalumab and durvalumab plus tremelimumab is associated with functional STK11 mutations in patients with non-small cell lung cancer and is reversed by STAT3 knockdown. Cancer Discov.

[56] Njatcha C, Farooqui M, Kornberg A, Johnson DE, Grandis JR, Siegfried JM (2018). STAT3 cyclic decoy demonstrates robust antitumor effects in non-small cell lung cancer. Mol Cancer Ther.

[57] Grada A, Otero-Vinas M, Prieto-Castrillo F, Obagi Z, Falanga V (2017). Research techniques made simple: analysis of collective cell migration using the wound healing assay. J Invest Dermatol.

