# Supplementary material for: Edible and cation-free kiwi fruit derived vesicles mediated EGFR-targeted siRNA delivery to inhibit multidrug resistant lung cancer
Source: J Nanobiotechnology. 2023 Feb 5;21:41. doi: 10.1186/s12951-023-01766-w (PMC9901103; doi:10.1186/s12951-023-01766-w)
Supplement: Supplementary file 1 — Additional file 1: Figure S1. TEM image of each band after sucrose density gradient centrifugation. Transmission electron microscope observation map of each ban. Figure S2. Average size change of the KEVs before and after incubation in 10% fetal calf serum at 37℃ for 24h. Figure S3. Viabilities of PC9-GR4-AZD1 cells after treatment with KEVs at different concentrations. Figure S4. Early and advanced STAT3 expression of NSCLC. Late-stage (stage III) display higher STAT3 expression level than early-stage (stage II) in the NSCLC. Figure S5. Characterization of siRNA loading efficiency. Figure S6. STAT3 expression in PC9-GR4-AZD1 cells. (A) The protein level of STAT3 was measured by Western Blot assay. (B) Analysis of the protein expression showed that STAT3/EKNPs treatment significantly reduced the expression of STAT3. Figure S7. Expression of EGFR in PC9-GR4-AZD1 NSCLC subcutaneous xenografts and paracancerous tissue. The protein level of EGFR was measured by Western Blot assay. Figure S8. STAT3 expression in PC9-GR4-AZD1 NSCLC subcutaneous xenografts. The protein level of STAT3 was measured by Western blot assay. Figure S9. Blood routine, liver function and kidney function of cationic liposome and STAT3 loaded cationic liposome treated mice, respectively. One-way ANOVA. ns, not significant. Error bars represent SEM (PBS group, n=3; Liposome group, n = 4; STAT3/Liposome group, n=5). [file 12951_2023_1766_MOESM1_ESM.docx]

**Edible and cation-free kiwi fruit derived vesicles mediated EGFR-targeted siRNA delivery to inhibit multidrug resistant lung cancer**


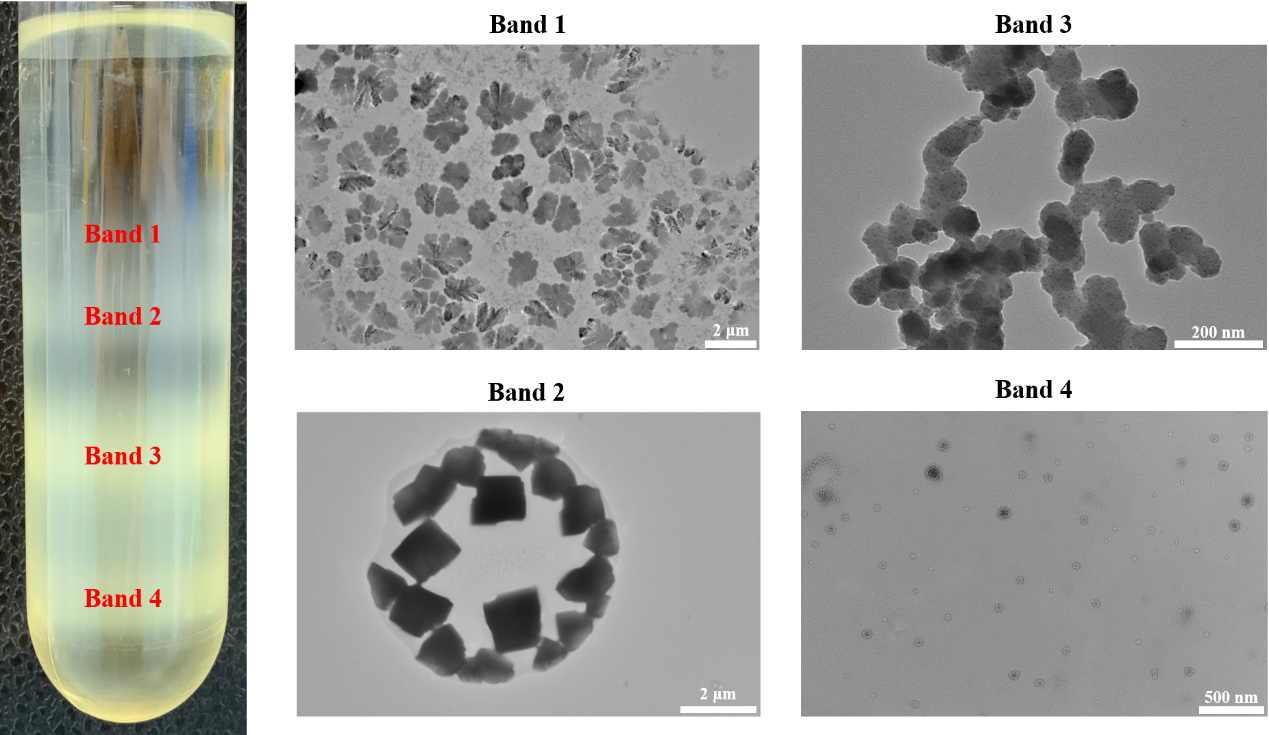


**Figure S1.** TEM image of each band after sucrose density gradient centrifugation. Transmission electron microscope observation map of each band.


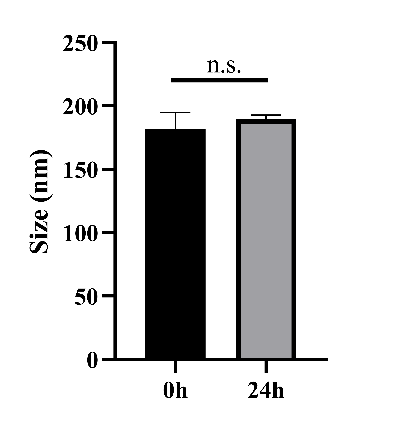


**Figure S2**. Average size change of the KEVs before and after incubation in 10% fetal calf serum at 37℃ for 24h.


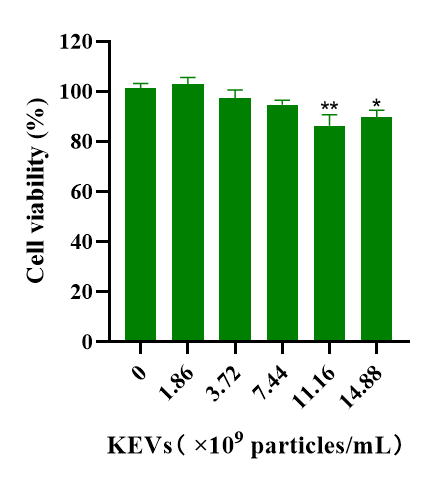


**Figure S3**. Viabilities of PC9-GR4-AZD1 cells after treatment with KEVs at different concentrations.


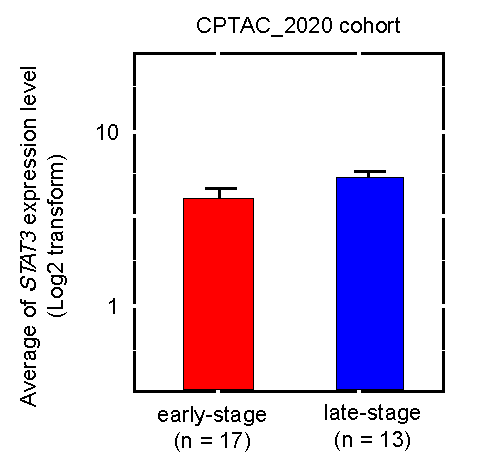


**Figure S4**. Early and advanced STAT3 expression of NSCLC. Late-stage (stage III) display higher STAT3 expression level than early-stage (stage II) in the NSCLC.


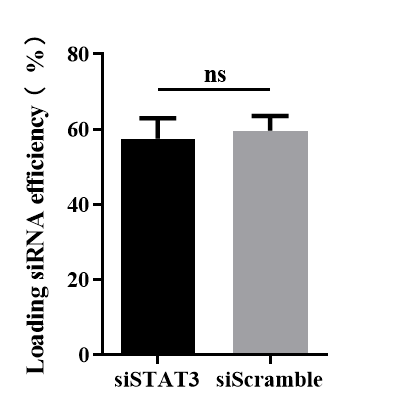


**Figure S5**. Characterization of siRNA loading efficiency.

**
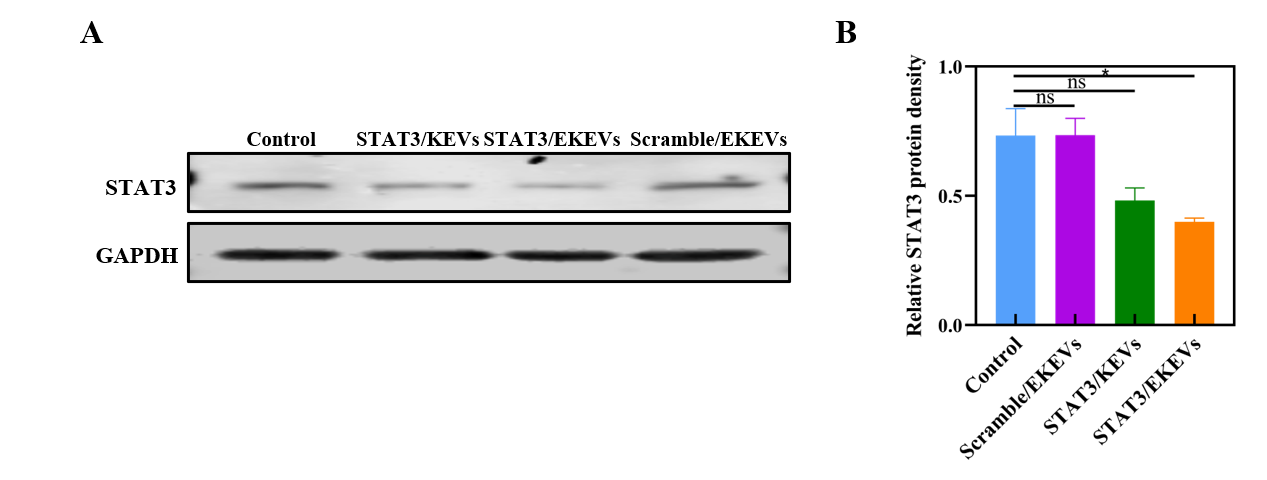
**

**Figure S6**. STAT3 expression in PC9-GR4-AZD1 cells. (A) The protein level of STAT3 was measured by Western Blot assay. (B) Analysis of the protein expression showed that STAT3/EKNPs treatment significantly reduced the expression of STAT3.


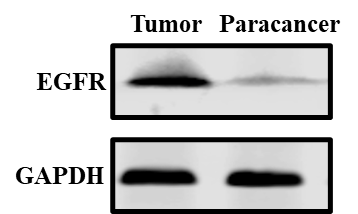


**Figure S7**. Expression of EGFR in PC9-GR4-AZD1 NSCLC subcutaneous xenografts and paracancerous tissue. The protein level of EGFR was measured by Western Blot assay.


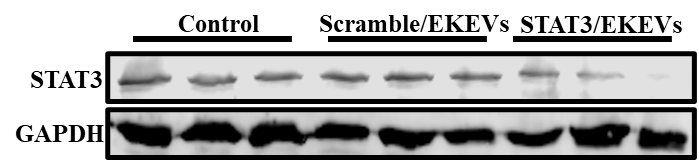


**Figure S8**. STAT3 expression in PC9-GR4-AZD1 NSCLC subcutaneous xenografts. The protein level of STAT3 was measured by Western blot assay.


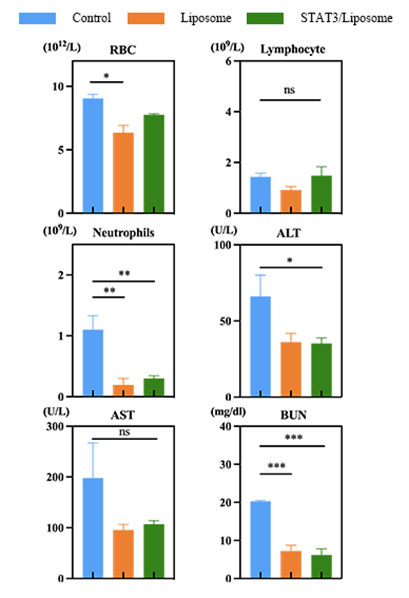


**Figure S9**. Blood routine, liver function and kidney function of cationic liposome and STAT3 loaded cationic liposome treated mice, respectively. One-way ANOVA. ns, not significant. Error bars represent SEM (PBS group, n=3; Liposome group, n = 4; STAT3/Liposome group, n=5).
